# Understanding Diabetes Through the Patients’ Lens: A Mixed-Methods Study on Experiences and Outcomes in Urban Areas of Rajasthan, India

**DOI:** 10.7759/cureus.81218

**Published:** 2025-03-26

**Authors:** Ramesh K Huda, Jayvardhan Singh, Ramesh Kumar Sangwan, Pankaj Kumar, Laxmikant Mandhana, Manoj Kumar, Bontha V Babu

**Affiliations:** 1 Department of Information Technology, National Institute for Implementation Research on Non-Communicable Diseases, Indian Council of Medical Research, Jodhpur, IND; 2 Division of Socio-Behavioural, Health Systems and Implementation Research, Indian Council of Medical Research, New Delhi, IND

**Keywords:** diabetes management, mix method, patient experience, self-management, type 2 diabetes mellitus, urban health

## Abstract

Objective: This study aimed to explore the diverse experiences and management strategies of individuals with type 2 diabetes mellitus (T2DM).

Methods: Participants diagnosed with T2DM were recruited from the Urban Primary Community Health Center, Jodhpur city, India. Quantitative data were collected, focusing on their diabetes management routines, interactions with healthcare systems, and personal and social challenges, followed by HbA1c and other vital parameters. Qualitative data included in-depth interviews. Thematic analysis was performed independently by two researchers using NVivo® software (Lumivero, Burlington, MA).

Results: One hundred forty patients (with a mean age of 56.81 ± 10.70 SD years, 62.9% men and 37.1% women) were recruited for the quantitative survey. The average waist circumference was 98.40 cm (SD = 10.53). A notable percentage of individuals (39.3%) had a BMI ranging from 25 to 29.9, and the average HbA1c level was 8.90% (SD = 2.08). The qualitative data analysis identified five themes, highlighting the need for improved diabetes management and health literacy for T2DM.

Conclusion: The diverse experiences of individuals with T2DM emphasize the need for improved management and health literacy. Addressing personal and social challenges and optimizing healthcare interactions can enhance patient outcomes, underscoring the importance of patient-centered interventions in diabetes care.

## Introduction

Type 2 diabetes mellitus (T2DM) is a severe and chronic condition marked by consistently elevated blood glucose levels. This occurs either due to insufficient insulin production or the body’s inability to use the insulin produced effectively. Diabetes impacts people of all ages, genders, and regions, contributing significantly to global mortality and morbidity [1-3]. As per the Global Burden of Diseases, Injuries, and Risk Factors Study 2019, diabetes ranked eighth in combined mortality and disability worldwide [1]. Approximately 537 million individuals across diverse demographics and nations lived with the condition in 2021. Projections indicate a continuous rise to approximately 643 million by 2030 and 10.9% to around 783 million by 2045 [4,5]. In India, one out of six adults suffer from the disease. Nearly 77 million people were estimated to have diabetes in 2019, and by 2045, that number is projected to reach over 134 million [6]. A recent study indicates that approximately 101 million individuals in India, constituting 11.4% of the nation's population, are currently affected by diabetes, while another study suggests that around 136 million individuals, equivalent to 15.3% of the population, may be living with prediabetes as of 2023 [7,8]. The increasing prevalence of diabetes presents significant challenges in terms of healthcare access, management practices, and health literacy. As the burden of diabetes continues to rise, it poses a considerable threat to public health, necessitating effective management strategies and interventions tailored to individual needs [9,10]. Effective management of T2DM involves addressing personal and social challenges, optimizing healthcare interactions, and promoting health literacy to improve patient outcomes.

While prior research has focused on the epidemiology and clinical outcomes of diabetes, there is a lack of comprehensive studies that integrate quantitative and qualitative insights to explore patients’ lived experiences, self-management strategies, and interactions with healthcare systems in an urban Indian context. This study fills this gap by providing a mixed-methods analysis of diabetes management, highlighting challenges and coping mechanisms, and offering insights into the role of health literacy, social support, and behavioral interventions in improving diabetes care.

By exploring patients' diverse experiences and coping strategies, the research will provide valuable insights into the personal and social challenges of individuals living with T2DM. Understanding these dynamics is crucial for developing patient-centered interventions that can enhance healthcare interactions and optimize management strategies for diabetes care.

## Materials and methods

Study design and setting

We conducted a mixed-methods study consisting of a quantitative component with a sample size of 140 T2DM patients, followed by a qualitative component involving in-depth interviews with 11 patients. The study was conducted at the Urban Primary Health Center (UPHC) in Jodhpur city, Rajasthan, India. Among the 17 Primary Health Centers (PHCs) in the Jodhpur Block, two PHCs (PHC-I and PHC-II) were randomly selected from geographically stratified groups, with the UPHC being one of these randomly chosen centers. Participants were eligible if they were 18 years or older, diagnosed with T2DM, had preintervention HbA1c levels of 6.5% or higher, owned a personal Android phone, could retrieve and read SMS in Hindi, and were available for the study duration. Exclusion criteria included clinical conditions that could interfere with participation, such as pregnancy, severe mental illness affecting cognition, or other serious illnesses.

Null Hypothesis (H₀)

There is no significant relationship between diabetes management practices like medication adherence, dietary habits, physical activity, healthcare interactions, and glycemic control (HbA1c levels) among individuals with T2DM in urban Rajasthan.

Ethical approval

The study received ethical clearance from the Institutional Ethics Committee of the authors' institute. Before participating, all patients provided written informed consent, which was secured after the study's objectives and methodologies were explained. All ethical procedures adhered to the principles outlined in the Declaration of Helsinki.

Study sample and data collection

The sample size was calculated based on an expected difference of 0.33 (equivalent to a 5% change) in HbA1c from the start of the study to six months later. Assuming a common SD of 0.65% and aiming for a statistical power of 90% to reject the null hypothesis with a two-sided significance level (α) of 0.05, 126 patients were needed. To account for a potential dropout of 14 patients, the plan was to recruit 140 patients. For the qualitative insights, 11 in-depth interviews were conducted and selected randomly from 140 patients. Interviews were continued until saturation was achieved, and no newer information was yielded regarding themes or information on the existing themes. Patients in this study were selected using purposive sampling, targeting individuals diagnosed with diabetes who regularly attended the UPHC. The list of diagnosed patients was obtained from the UPHC, and participants meeting the inclusion and exclusion criteria were recruited accordingly.

Consenting patients first underwent HbA1c testing, followed by vital parameter measurements. A pretested and validated questionnaire was administered to gather information on sociodemographic characteristics (such as age, gender, education, and occupation), disease management (including the year of T2DM diagnosis and chronological healthcare itineraries), etc. This was followed by in-depth interviews (IDIs) conducted in either Hindi or English, based on the patient’s choice, audio-recorded on the hospital premises to ensure consistency. IDIs were conducted to gain insights from patients on diabetes management, including diagnosis, medication adherence, self-monitoring, diet, exercise, healthcare visits, and perceived barriers to seeking care (refer to the Appendix). These interviews took place quietly at the hospital and lasted about 20-30 minutes. All interviews were audio-recorded. Interviews were transcribed, and primary coding was performed by two investigators, who then condensed the codes into themes or categories. The author translated and transcribed these recordings verbatim into English. The study recruited participants from November 2023 to February 2024.

Data analysis

We performed descriptive and inferential statistical analysis of sociodemographic, lifestyle, and behavioral parameters as physical and biochemical variables. Concerning the quantitative data, an inferential analysis through the chi-square test was conducted using Statistical Package for the Social Sciences version 28 (IBM Corp., Armonk, NY). After becoming thoroughly acquainted with the content, both researchers independently coded the qualitative scripts using NVivo 14 (Lumivero, Burlington, MA). A generic thematic analysis approach facilitated the organization of codes into themes across several iterations. Any discrepancies in coding were collaboratively resolved through discussions involving a third author when necessary. To ensure consistency and reduce potential bias, emergent themes were extensively reviewed and refined through discussions among all authors.

## Results

The study population is predominantly older, with a mean age of 56.81 ± 10.70 years and a significantly higher proportion (70%) in the ≥51 years category (p < 0.005), suggesting a greater burden of health risks in older individuals. Occupation and education levels do not show significant associations (p > 0.05), indicating that these factors may not strongly influence health outcomes in this sample. However, waist circumference (98.40 ± 10.53 cm) and BMI (28.36 ± 4.72) are significantly associated (p < 0.001), highlighting obesity as a potential concern. Blood pressure and heart rate values, though elevated (mean systolic: 142.80 mmHg, diastolic: 83.30 mmHg), do not show significant variations (p > 0.05), suggesting hypertension is prevalent across the group. Smoking prevalence is relatively low (7.1%, p = 0.085), but the mean HbA1c of 8.90 ± 2.08 indicates poor glycemic control, suggesting a high burden of diabetes in the population (Table 1).

**Table 1:** Association of demographic characteristics with HbA1C (n = 140) ^*^p value of <0.005 is significant

Variables	Categories	Mean ± SD	n (%)	p value
Age	≤30 year	56.81 ± 10.70	1 (0.7%)	<0.005^*^
	31-50 year		41 (29.3%)	
	≥51 year		98 (70%)	
Sex	Male	-	88 (62.9%)	0.530
	Female	-	52 (37.1%)	
Occupation	Employed	-	77 (55%)	0.927
	Unemployed	-	42 (30%)	
	Retired	-	21 (15%)	
Education	No formal education	-	28 (20%)	0.736
	Up to 10th of schooling	-	50 (35.8%)	
	Up to 12th of schooling	-	17 (12.1%)	
	Graduation	-	28 (20%)	
	Postgraduation and above	-	17 (12.1%)	
Waist circumference (cm)	-	98.40 ± 10.53	-	<0.001^*^
Body mass index	<18.5	28.36 ± 4.72	2 (1.4%)	<0.001^*^
	18.5-24.9		34 (24.3%)	
	25-29.9		55 (39.3%)	
	>30		49 (35%)	
Blood pressure (mmHg)	Systolic	142.80 ± 17.96	-	0.948
	Diastolic	83.30 ± 9.27	-	0.743
Heart rate	-	86.07 ± 14.16	-	0.341
Smoking habit	Yes	-	10 (7.1%)	0.085
HbA1c (%)	-	8.90 ± 2.08	-	-

Most patients (114, 81.4%) reported no history of chronic disease. Most patients (102, 72.9%) visited outpatient clinics monthly, with 77 (55%) checking their blood sugar levels at health centers and 45 (32.1%) doing so at home. Only 55 (39.3%) consistently recorded their results, while 50 (35.7%) did not record them at all. Regarding medication adherence, 35 (25%) sometimes forgot to take their doses, and 13 (9.3%) were occasionally careless. Awareness of diabetes complications was high (110, 78.6%), but only 27 (19.3%) were knowledgeable about a healthy diabetic diet. Regular snack consumption was reported by 90 patients (64.3%), and 109 (77.9%) exercised daily. However, 118 (84.3%) did not engage in strenuous activity, despite 127 (90.7%) being aware of its benefits (Table 2).

**Table 2:** Lifestyle and behavioral characteristics of participants (n = 140)

Variables	Categories	n (%)
History of chronic diseases	Yes	26 (18.6%)
	No	114 (81.4%)
How often do you visit the hospital as an outpatient?	First time	2 (1.4%)
	Weekly	3 (2.1%)
	Monthly	102 (72.9%)
	Upon need	33 (23.6%)
Where do you usually check your blood sugar?	Health center	77 (55%)
	Lab	12 (8.6%)
	Glucometer at home	45 (32.1%)
	Do not check	6 (4.3%)
Do you record your blood sugar results?	Always	55 (39.3%)
	Usually	22 (15.7%)
	Sometimes	13 (9.3%)
	Not recording	50 (35.7%)
Do you sometimes forget to take your medication/insulin?	Yes	35 (25%)
	No	89 (63.6%)
	Not taking any treatment	16 (11.4%)
Excluding times you forget, are you sometimes careless taking your medicine/insulin?	Yes	13 (9.3%)
	No	111 (79.3%)
	Not taking any treatment	16 (11.4%)
Are you aware of diabetes complications?	Yes	110 (78.6%)
	No	30 (21.4%)
Are you aware of the healthy diet for diabetics?	Yes	27 (19.3%)
	No	113 (80.7%)
Do you regularly eat snacks between main meals?	Always	90 (64.3%)
	Usually	13 (9.3%)
	Sometimes	17 (12.2%)
	Do not eat	20 (14.3%)
Do you regularly exercise any physical activity?	Daily	109 (77.9%)
	Weekly	2 (1.4%)
	Upon need	6 (4.3%)
	No exercise	23 (16.4%)
Do you exercise strenuously?	Always	5 (3.6%)
	Usually	4 (2.9%)
	Sometimes	13 (9.3%)
	No exercise	118 (84.3%)
Are you aware of the benefits of exercise for diabetics?	Yes	127 (90.7%)
	No	13 (9.3%)

The results from the in-depth interviews are presented in Table 3, organized into key themes and subthemes.

**Table 3:** Major themes and subthemes from in-depth interviews of diabetes patients

Subtheme	Key findings	Representative quotes
Theme 1: Diabetes management and control. This theme highlights the patients' proactive efforts in managing their diabetes through consistent medication adherence, regular home blood sugar testing, and periodic comprehensive assessments at medical facilities. Patients emphasized the importance of healthy and active eating habits, recognizing that these practices are essential for effective diabetes control		
Medication management	Patients exhibit strong medication adherence through consistent routines and attention to timing. They follow specific instructions regarding when to take their medicines in relation to meals, ensuring optimal absorption and effectiveness	P1: I take my medicines in the morning and in the evening. I also take my medicines 10-15 minutes before eating. I take my medicines on time
		P6: I take medicines 20-25 minutes before eating food. I take medicines half an hour after taking BP medicine. These medicines have been going on for six to seven years
Dietary management	This highlights the essential role of dietary choices in diabetes management. Their preference for millet-based porridge without added Sugar demonstrates an understanding of the importance of low-glycemic foods for better blood sugar control	P1: I used to eat roasted food, Sabudana. Yes, it is sweet. Makhana. I used to get it separately. I used to eat roasted food. I used to eat it. Then, I used to eat Salad in a box. You can eat it in the middle. Then, I used to eat liquid food. Okay. I used to eat less roti and more curd, buttermilk, etc.
		P3: By eating healthy food, it depends on the food. For food, like in the morning, we had milk porridge, and we didn't add sugar to it. And in porridge, if we take the mixed (millet)one, then we are in control of it
Physical activity and exercise	This subtheme underscores the patients' dedication to incorporating regular physical activity into their daily routines, with many mentioning the importance of morning walks	P4: There is a round of 800 meters; I used to do five rounds of it. I used to go for a walk at 5.30 in the morning and come back at 7 in the morning
Monitoring and health tracking	By utilizing a combination of home blood sugar testing and scheduled visits to medical facilities, individuals ensure that they stay informed about their health status. This proactive approach allows for timely adjustments to their treatment plans based on real-time data, ultimately enhancing their ability to control blood sugar levels and mitigate potential complications	P1: I do a sugar test at home. For HbA1c, in the hospital. Once every six months, a whole test should be done
		P3: I get it done in MDM every year. Yes, once a year, we also do it, and there is a fasting instrument at home, so we fast every Sunday. Fasting Blood tells us our level and whether the medicine is working properly or not
		P 12: Government trainer's hospital. I get it done at Nehru Hospital three to four times a month. I get it done at home as well. Once in 7-10 days
Theme 2: Social and emotional dynamics. This theme examines the interplay between social support and individuals' emotional challenges in managing diabetes. Patients noted that family routines have adapted to support diabetes management, with shared meals incorporating healthier options like green vegetables and mixed grains. Emotional support from family members is vital, as it provides motivation for exercise and reinforces healthy eating habits. Patients also experience significant emotional stress related to their condition, including feelings of depression, anxiety, and tension over fluctuating blood sugar levels		
Family and social support	This section highlights the vital role of family and social support in managing diabetes. Patients shared how their families have embraced healthier lifestyles, with partners motivating them to exercise and adopt better dietary habits. This collective approach promotes accountability, strengthens family bonds, and significantly enhances the effectiveness of diabetes management	P1: My husband motivates me, so I mostly do (evening exercise). Because of me, they have also changed their lifestyle. They used to take me to the park to exercise. They changed my diet
		P2: We both are sugar patients. I have a son. So, he also eats corn porridge with me in the evening. He also eats it. I tell him, Son, look, I have bought it. In the future, you will also have it. So, you control it from now on. I add sugar to the juice I make. I take it out for both of us. So, and like, this is mixed wheat bread, so I feed it too. I won't make it separately for you. It will be made together. And, like, there is rice, porridge, vegetables, and potatoes. He sometimes says a lot about Khichdi. So, I eat it twice a month. I feed him, too
		P3: Yes, we all, meaning three brothers and sisters, all three of us follow the same diet, and there is no problem; it is necessary that, like sugar-free tea, they add sugar separately
Emotional and psychological responses	This subtheme addresses the emotional and psychological challenges faced by individuals managing diabetes, highlighting the impact of health-related stress and life circumstances on mental well-being. Factors such as family issues, unemployment, and general life pressures contribute to feelings of confusion and depression. There is a need for support systems that address both the emotional and physical aspects of diabetes management	P3: I mean, I am depressed a lot, sometimes it is unwanted, sometimes it is children's tension, sometimes there is no work, so, with that, the mind is disturbed, so the mind becomes very puzzled
		P6: I used to get tense if it was high. I used to get tense if it was low. I used to worry about it. I used to control it. BP and sugar were both high. If it was low, I used to worry about it. I used to feel dizzy. I used to feel dizzy from head to toe. So, the doctor started giving me sleeping pills. I said that I would take one pill and suppress it
Coping strategies and resilience	This sub-theme highlights the adaptive strategies patients use to manage their diabetes effectively. Many show resilience by adhering to dietary plans that help control their blood sugar levels. Practical coping strategies include carrying sweets for quick relief during low blood sugar episodes and reflecting advice from healthcare providers. Additionally, patients demonstrate an intuitive understanding of their bodies, recognizing physical cues like thirst and dry lips that indicate changes in blood sugar levels. Patients develop resilience through dietary adherence, practical strategies, and heightened self-awareness in managing their diabetes	P2: I am happy with my diet plan because my sugar level has been controlled. I have been following it for two years. I do follow it when I go out. But mostly, I follow it at home. I used to like sweet food, but now I avoid it. Now you don't like sweet food. I don't like it much. In the beginning, it was different in taste. But now I am used to it. Like, I mostly drink Glucon-D. I drink lemon water, etc. I ate a little sweet at that time. Whether it was chocolate or anything else, I kept it in my bag. Our doctors also say this. If you feel like having low Sugar, then eat a little sweet
		P6: If I feel a lack of sugar, then I eat something first and then see. I do morning walks. I feel that my sugar level is increasing. So, at that time, I reduced my food intake. And today, I feel that my sugar level is decreasing. So, I feel thirsty; my lips will dry. So, these automatic sensors of the body tell me what changes are occurring in my body. So, in the recording of that, I follow my body
Theme 3: Health literacy and education. This theme offers patients an active role in seeking information about diabetes management from diverse sources, including YouTube, healthcare professionals, and digital tools. Patients highlight the significance of diet, exercise, and blood sugar monitoring in managing their condition. Many explore alternative treatments, such as Ayurvedic and Homoeopathic medicines, alongside traditional remedies while recognizing the necessity of allopathic medications for effective diabetes control. This proactive approach to health literacy empowers individuals to make informed decisions about their diabetes management		
Education and information seeking	This subtheme highlights how individuals actively seek dietary information to manage their diabetes. Patients emphasize reducing refined carbohydrates like wheat and incorporating healthier alternatives such as whole grains and millet due to their lower glycemic index and higher fiber content, which help stabilize blood sugar levels. Many individuals obtain dietary advice from various sources, including YouTube and dieticians, who recommend foods like idly, khaman, and dalia while advising against excessive gluten consumption	P2: I have heard this only. When I watch on YouTube, wherever I read, I read this only. In terms of glucose, most people in India say they should stop eating wheat. So, eat millet porridge and corn porridge; eat more of this. Eat more mixed wheat bread. Don't eat wheat; reduce it. And don't drink juice, eat fruits
		P3: The dietitian told me to add this salad. The dietitian told me that, in the morning, you have idly, khaman, dalia, and milk. Take these things, mostly; I mean, you don't have to take gluten too much. So, that also increases Sugar. Okay. So, they told me to take this mixed one, Bajri, Jowar, and these five, mix them, and make dalia
Alternative treatments and beliefs	Focusing on how individuals adopt a holistic approach to diabetes management by integrating traditional and alternative treatments with lifestyle changes. Patients express confidence that methods like Ayurvedic practices and specific dietary choices can effectively control glucose levels, preventing harm associated with uncontrolled diabetes. Lifestyle adjustments highlight their commitment to managing diabetes comprehensively	P3: If you take Ayurvedic medicines or do some diet work, then you will get this under control. People say that as much as you keep it under control, it will not harm you. If you don't keep this sugar under control, then it will harm you
		P5: I take Neem Leaves daily in Ayurveda. Apart from that, I do workout in the morning
		P6: These are medicines that control sugar. Now, I take 10 drops of homeopathic liquid. I am taking that. I am taking five types of lentil rotis. I don't take in sweets, I don't eat rice, I don't eat potatoes
Theme 4. Healthcare interaction. This theme highlights patients' varied experiences in their diabetes diagnosis and interactions with healthcare providers. Patients seek information from hospitals, physicians, and alternative sources, reflecting their desire for comprehensive understanding and support. Regular follow-ups, medication adjustments, and blood glucose monitoring are common; some use home glucometers, while others rely on clinic visits. Patients navigate the healthcare system by consulting specialists to manage diabetes and other health conditions		
Diagnosis of the condition	This sub-theme focuses on the journey toward a diabetes diagnosis, highlighting the progression from experiencing symptoms to receiving formal recognition of the condition. Patients describe incidents where diabetes was identified during consultations for unrelated health issues, such as urinary infections or general health check-ups	P1: Four years ago, I had a urinary infection. When I consulted a gynecologist, she diagnosed me with diabetes. I am taking medicines from Goyal Hospital
Healthcare system navigation	This subtheme addresses the challenges patients encounter in navigating the healthcare system for diabetes treatment. Patients often face a choice between continuing care at their current facility or seeking treatment elsewhere, with many preferring to stay due to the difficulties of accessing care at hospitals. Patients express concerns about travelling to different facilities, citing energy constraints and the discomfort of waiting without food	P3: Initially, I came to know about diabetes in 2016. At that time, I used to feel hungry and dizzy. BP also used to be a problem, so I didn't pay much attention to diabetes, which can also be there. Then, when I went for a meeting at PHC, Madam checked me. Madam was there, and she checked me and told me that I have diabetes. She recommended I either take treatment from MDM or do regular treatment from here
		P7: I have shown the diseases in the mouth or something else. Here I get the medicine, it is the best thing. And to go to Paota Hospital, I don't have so much energy, I have to go hungry. There, they call the doctor three to four times
Theme 5. Challenges in diabetes management and health concerns. This theme focuses on the various barriers patients face in effectively managing their diabetes. Key challenges include busy schedules that lead to forgetfulness in medication and dietary adherence, as well as fear of medication side effects. Many struggle with maintaining regular exercise and monitoring their blood sugar levels, compounded by financial constraints that limit access to necessary resources. Additionally, long-term health concerns and anxiety about potential complications add to the emotional burden. Overall, this theme highlights the complex interplay of practical, financial, and emotional factors that impact diabetes management		
Barriers to effective management	This sub-theme outlines the various obstacles patients encounter in managing diabetes. Time constraints from family and work commitments hinder their ability to adhere to healthy routines. Fear of blood glucose monitoring through pricking causes some to avoid checking their levels, while forgetfulness in taking medications is common, particularly among those facing fatigue from night shifts. Physical symptoms like tiredness and foot pain further reduce motivation for exercise	P1: I don't get time in the morning. My daughter goes to school. I don't get time in the morning. I have to eat food. I have to come on duty. I have to reach here by 8 o'clock
		P2: I am afraid. I am busy, and I have less time. I am busy; I am in the field, and I have to do reporting and a lot of work. Sometimes, I forget to do it here, as well. I am afraid of a prick
		P3: Sometimes, I forget to take medicines, and it happens frequently now that I have developed the habit of forgetting. My memory is very weak, and one more thing is that after two-night shifts on duty, I feel sleepy
		P9: I have to go around in the garden in the evening. I believe exercise and work are the same
		P11: I feel lazy, I feel tired, I have pain in my feet, I can't walk
Long-term health concerns	This subtheme explores patients' anxieties regarding the long-term management of diabetes and the potential consequences of medication use. Many express fears about becoming reliant on medications and question the feasibility of controlling diabetes without them. Conflicting views on the effectiveness of these treatments contribute to their uncertainty. Patients also worry about possible side effects, with some believing that prolonged use of diabetes and blood pressure medications could weaken their bodies. This reflects a broader concern about how diabetes impacts overall health and quality of life	P2: I have heard from people. So, I was also a little scared that if I don't take medicines, how can I get rid of diabetes? Sometimes, when I take medicines, people say that my diabetes is very low
		P6: The reason for stopping medicine is that someone told me that your body parts will become weak. If you take BP and sugar medicines continuously, your body parts will become weak. These medicines are not sugar medicines from day to day
		P7: My husband also had it. That time, I didn't understand so much. But now, when I have it, he says, neither can we eat nor can we drink, which means this disease makes the body hollow inside

## Discussion

This study explores the multifaceted dimensions of T2DM management among patients, emphasizing medication adherence, dietary habits, physical activity, monitoring, coping strategies, social support, health literacy, healthcare interactions, and the barriers to managing the condition.

Anthropometric and clinical measures revealed that the average waist circumference was 98.40 cm, and a significant proportion of patients had a BMI between 25 and 29.9 (39.3%). These values indicate a high prevalence of overweight and obesity among patients, which are known risk factors for poor glycemic control and diabetes complications [9].

Our findings highlight a high level of adherence to prescribed medication schedules among patients, with structured routines and regular blood sugar monitoring being central to their management strategies. This is consistent with previous research, which suggests that medication adherence is critical to effective diabetes management and improved glycemic control [10]. Patients in our study reported making significant dietary adjustments, focusing on healthy eating habits to control blood sugar levels. This underscores the role of dietary management in achieving glycemic targets [11]. Many patients also reported regular physical activity as a key component of their diabetes management. This finding is consistent with the literature, which emphasizes the role of exercise in improving insulin sensitivity and glycemic control [12]. In the current study, patients reported that physical activities like yoga and morning walks helped them manage diabetes. Similar results were found by Umpierre et al., indicating that 150 minutes of structured exercise resulted in a decline in HbA1c levels [13].

Frequent monitoring of blood glucose levels at home and in healthcare facilities was a common practice among patients. This practice is crucial for managing T2DM effectively and preventing complications [14]. The study underscores the importance of family and social support in diabetes management. Family members often adjust their routines to support the patient, consistent with research indicating that social support positively impacts diabetes outcomes [15]. However, patients also reported significant emotional and psychological stress related to their condition, echoing findings from other studies that highlight the psychological burden of diabetes [16].

Patients actively sought information from various sources to manage their diabetes better. This behavior aligns with findings that higher health literacy is associated with better diabetes self-management and outcomes. Patients with inadequate health literacy were less likely than those with adequate health literacy to achieve tight glycemic control [17]. In this study, people reported using social media platforms to increase their knowledge about diabetes management. Another study reported that exploring alternative treatments, such as natural products and yoga therapy, reflects a broader trend of patients seeking complementary and integrative approaches to healthcare [18]. Patients' experiences with healthcare interactions varied, with many reporting incidental diagnoses and seeking information from multiple sources. This variability is consistent with findings that patient experiences and satisfaction with healthcare services can significantly impact diabetes management [19]. Regular follow-ups and adjustments in treatment plans were common, aligning with the need for continuous medical supervision in diabetes care. The present study also showed treatment adherence [20].

Patients faced several barriers, including low education levels, advanced age, low health literacy, and the spread of misinformation. These factors affected patients' motivation to engage in their care actively and led to resistance to intensifying treatment and achieving their clinical management goals [21]. Financial constraints and lack of time further complicated adherence, highlighting the socioeconomic factors influencing diabetes management [22,23]. Similar findings were reported due to the patient's low income and long-term health concerns, and fear of medication side effects was significant among patients with diabetes.

The responses reveal several gaps in practices related to diabetes management, particularly concerning dietary choices, mental health, and adherence to conventional and alternative treatments. Information from social media platforms may lead to misinformation or inconsistent dietary habits. Additionally, while some patients actively engage in alternative treatments like Ayurveda and Homeopathy, there is a lack of integration with conventional medical advice, which may result in gaps in overall treatment effectiveness. Mental health issues, such as stress and anxiety related to blood sugar fluctuations, are also prevalent. Yet, patients do not mention using any mental health resources or strategies to manage these concerns. This oversight highlights a need for a more holistic approach to diabetes management.

Information technology tools can be highly beneficial in addressing the diverse needs and gaps in diabetes management, particularly in medication adherence, diet, physical activity, mental health, and alternative medicine approaches. For medication management, mobile apps and SMS reminders can ensure timely intake of treatments and provide tracking. These tools can also provide alerts to avoid certain foods like gluten, which some patients associate with increased blood sugar levels. Stress, in particular, can affect blood sugar control, as noted by patients who experience anxiety over fluctuating glucose levels.

The study has significant strengths, providing a detailed understanding of the lived experiences of diabetes patients and covering various management dimensions. However, a limitation is the potential for selection bias due to the smaller representative sample size, specifically from urban areas, which reduces the generalizability of the study findings.

## Conclusions

This study highlights the multifaceted challenges of T2DM management among urban patients in Rajasthan, emphasizing gaps in health literacy, lifestyle modifications, and access to quality healthcare. The findings indicate that while many patients engage in daily physical activity, the intensity remains insufficient, and medication adherence is inconsistent. Poor glycemic control, coupled with a high prevalence of obesity and limited awareness of healthy dietary practices, further complicates disease management.

Beyond the clinical aspects, diabetes care is deeply influenced by social and psychological factors. Emotional distress, anxiety, and misinformation significantly impact patients’ self-management strategies. While family and social support are crucial in reinforcing positive health behaviors, financial constraints and limited healthcare access often hinder optimal disease control. Many patients rely on alternative treatments without professional guidance, reflecting the need for integrated healthcare approaches that combine conventional and complementary medicine.

The study underscores the necessity of patient-centered interventions that go beyond medical treatment. Strengthening health education programs, promoting culturally appropriate lifestyle modifications, and integrating technology-driven solutions such as mobile health applications and digital reminders could enhance adherence and self-care. Addressing psychosocial challenges through mental health support and community-driven awareness initiatives will be essential in improving long-term outcomes. Given the growing burden of T2DM, a multidisciplinary approach that bridges medical, behavioral, and technological solutions is critical to ensuring better diabetes management and improving the overall quality of life for affected individuals in urban India.

## Appendices

**Table 4:** Interview guide This interview guide was developed by authors and was validated by domain experts. It is an original creation and is used in this study with due credit to the authors

Variables	Questions	Probe
Introductory question	Let us talk about your diabetes, how you were diagnosed with diabetes and where you go to get the information about your diabetes.	-
	How do you take care of your diabetes?	-
Practices toward diabetes medication	When do you take your medicine?	Do you understand how to follow your medication?
	How do you identify which of your medications are used to treat diabetes?	-
	Do you ever decide not to take care of your diabetes?	Do you worry about side effects? Regular exercise and strict dietary precautions replace medication requirement busy, forgetful tired of taking medicines
Knowledge and practices toward self-monitoring of blood glucose	What blood sugar level has your doctor suggested is good for you?	-
	How do you get your blood glucose level checked?	Home, clinic, frequency, and glucose monitor
	Do you face any problems when checking your blood sugar?	-
	What do you do when you feel shaky, hungry, sweaty, thirsty, tired, and weak, or what do you do when you do not feel well?	-
Knowledge and practices toward diet management	What do you think about controlling your blood sugar through healthy food choices?	What do you mean? In what way will it make it easier for you?
	What food choices would make a difference in your blood sugar control?	-
	Was current knowledge about the diet plan obtained from a physician or a dietitian recommended by him?	-
	How have you and your family members changed your daily routine to follow it?	-
	What makes it challenging to adopt a diet plan?	Favorite foods, job nature, affordability, and social traditions
Knowledge about exercise and barriers to it	What sort of physical activity is recommended to you by your physician? Are you following it?	-
	Are you facing any problems for this? (What kind?)	Regularity confusing their job exertion as a replacement for regular exercise influence of weather
Continuity of care	For the following questions, I want you to think about those visits to your doctor related to your diabetes. If you saw a doctor for both your diabetes and another condition, that would still count as a visit related to your diabetes. What makes you decide when you should go to visit your doctor for follow-up care?	What types of health problems make you visit the primary care doctor? How often do you visit your primary care doctor for regular checkups? How often do you visit other healthcare providers because your primary care doctor has referred you? How often does your primary care doctor ask about other medications you are taking?
